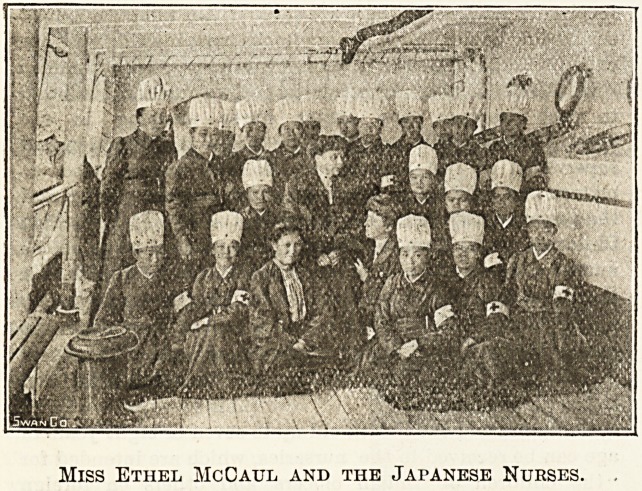# The Hospital. Nursing Section

**Published:** 1904-12-10

**Authors:** 


### The Hospital.

### Nursing Section.

Contributions for this Section of "The Hospital" should be addressed to the Editor, "The Hospital," Nursing Section, 28 & 29 Southampton Street, Strand, London, W.C.

No. 950.—Vol. XXXVII. *SATURDAY, DECEMBER* 10, 1904.

### Notes on News from the Nursing World.

### OUR CHRISTMAS DISTRIBUTION.

We remind our readers that the last day for sending in parcels for distribution at Christmas is Saturday, December 17th, and we hope that a large number of useful contributions will be received in the interval between this and that date. The whole of the articles forwarded will be on view at the offices of The Hospital on Tuesday afternoon, the 20th. Nurses or any contributors who desire to see for themselves the garments which have been forwarded to us will be welcome any time between 3 and 5 p.m. We have to acknowledge the receipt of a large parcel from 545 Policy Number, Royal National Pension Fund ; and we also learn that one of the parcels which was sent anonymously is from Miss Knight, 42 Cluson Road, Tufnell Park, N. All contributions should be addressed to the Editor, 28 & 29 Southampton Street, Strand, London, W.C., and shonld have "Clothing Distribution" writen outside them.

### THE DEPRESSION IN SOUTH AFRICA.

We publish to-day another warning to nurses, written by a correspondent whose reliability can be depended upon, advising them not to go out to South Africa, or if they go to be prepared to sink a hundred pounds while they are waiting for employment. This time the warning comes from Johannesburg, where streets were once supposed to be paved with gold, and is from a private nurse who herself has undergone the experience from which she wishes to save her sisters in England. The fact that within three months we have received two such emphatic proofs of the difficulty experienced by people with reduced incomes in paying the high fees which a nurse is compelled to charge in South Africa, is sufficient evidence of the depression.

### NEW NURSERIES FOR INFANTS.

The opening of a new undertaking in connection with the well-known Norland Institute is reported in another column. As a practical training school for the Norland probationers the founding of the nurseries is an excellent idea. But, while fully acknowledging the boon which these nurseries may be to children exceptionally situated, it is obvious that the Institute, in receiving babies from a month old, is taking upon itself a grave responsibility. Moreover, it seems to us that people able to afford the fees will not often care to send such very young children to the nurseries. With regard to the equipment most of the arrangements seem excellent, but the use of gas-stoves in the night nurseries is from every point of view a mistake. They should be removed at once and the ventilation improved.

### A CRY FROM INDIA.

Mrs. Creighton states that she has practically advertised in vain for English nurses to go out to India and undertake the duty of helping to train native women as nurses. We are very sorry to hear it, and we are sure that there are many nurses in this country who are ready to respond to the cry of the women in India which Mrs. Creighton says is sounding in our ears. This mention of the need will probably bring volunteers for a noble and necessary work.

### THE WORK OF THE IPSWICH NURSES' HOME.

Three branches of work are undertaken in connection with the Ipswich Nurses' Home, and the financial position of each was indicated at the annual meeting the other day. On the district branch there was a deficit of £231 15s. 4d. for the 12 months ; on the cottage branch, a deficit of £26 7s. 5d. ; and as to the private branch, the chairman said that it was, of course, self-supporting. No statement as to the profit, or whether there was any, was forthcoming. We notice, however, that the chairman said that the condition of the funds of the home was not serious enough to render the committee over-anxious, and that he seemed content to lay stress on the importance of getting back the £50 a year which the Corporation had withdrawn. But even if the £50 should be restored, there is a good deal of lee-way to be made up. Some of it, the committee think, may be obtained by levying a charge of 1d. a visit on the sick poor, but it is considered essential to increase the number of the district staff, and therefore the expenses materially. The new lady-superintendent, who went to Ipswich from Guy's Hospital last January, seems to be managing the work admirably, but the townspeople will need to subscribe much more liberally in order to put the institution on a sound basis.

### SEVEN YEARS AS QUEEN'S NURSE.

Quite the most interesting feature at the annual meeting of the Warrington District Nursing Association was a paper read by the superintendent after the report—which shows that nearly 19,000 visits were paid and that there was a deficiency of £25 on the working of the Association—had been adopted. Miss Whitfield, who has been superintendent from the outset, referred to the work of the late William Rathbone, a pioneer of district nursing, and affirmed that what Mr. Rathbone was to Liverpool Mr. Monks has, to a very great extent, been to Warrington. In the course of her survey she dissipated the notion that one of the difficulties of starting an organisation is that the poor resent the visits of the nurses as an intrusion. From the first, she said, there was never the slightest difficulty in this respect. Rarely had there been a repulse on the part of the patients, though a few stray aged persons, who had not been thoroughly washed since infancy and had no mind to undergo the process, objected, and a little boy, who knew that nurse's visit surely meant pain for the moment, had manifested his disapproval by repeating again and again the fact that "he didn't want that girl with a big pinny on." The history which the superintendent was able to give entirely justifies her remark that in seven years she and her staff find themselves stewards of a large trust; while the esteem in which they are held in Warrington proves that they are fully alive to their responsibility.

### THE CENTRAL MIDWIVES BOARD AND POOR-LAW INFIRMARIES.

An adjourned meeting of the Central Midwives Board was held on Thursday last week. The secretary stated that he had received a letter from the secretary of the Royal British Nurses' Association intimating that Mrs. Josephine Latter had been chosen to succeed Miss Oldham as representative of the association on the Board. Coombe Lying-in Hospital, Dublin, Guy's Trained Nurses' Institution and East End Mothers' Home were approved for the training of midwives under Section C of the rules. Some discussion arose over the question of a Poor-law Infirmary, and Miss Wilson pointed out the entirely different circumstances which had to be taken into consideration in these cases. She reminded the Board of the need for particular inquiry into the management and working of Poor-law institutions, and said that the general and lying-in wards were not seldom undesirably near each other. A member suggested that here was a case for inspection; and the Chairman, remarking that the points raised were of great importance, proposed that specially-framed questions should be addressed to Poor-law infirmaries with the view of finding out where any interchange of nurses was allowed between the general and the lying-in wards.

### HOSPITAL FOR INVALID GENTLEWOMEN

On Tuesday and Wednesday a sale of useful and fancy articles will be held at the Hospital for Invalid Gentlewomen, 90 Harley Street. It will be open in the afternoon from 2.30 P.M. The hospital founded by the late Viscountess Canning, whose first lady superintendent was Miss Florence Nightingale, must always have a claim upon the sympathy and support of the public. Two years ago Miss Nightingale herself wrote, "I ask and pray my friends who still remember me not to let this truly sacred work languish and die for the want of a little more money." Funds are now urgently needed to provide for the removal of the hospital, and all who make purchases at the bazaar will help one of the most valuable institutions in the capital.

### LARGE DEFICIT AT GLASGOW.

At the annual meeting of the Glasgow Sick Poor and Private Nursing Association, two statements were made which do not reflect credit on the people of the great city on the Clyde. The expenditure exceeded the income to the extent of £724; and the general subscriptions, amounting to £825, were the contributions of less than 400 people and firms. This means that a great many individuals and firms did not contribute at all. Possibly, as in most cases where district nursing and nursing for payment is carried on by the same association, there may be an idea that the organisation ought to be self-supporting. This, however, the collectors should take care to dissipate. In fact no fewer than 2,845 cases were treated in the district branch, and these figures should suffice to establish the claim of the movement on the pockets of the charitable. It is satisfactory to note that there has been an increase in contributions from public works.

### QUEEN'S NURSES AT READING.

At the seventh annual meeting of the Queen Victoria Nursing Institute, Reading, it was announced that owing to the efforts of the Ladies' Auxiliary the subscriptions showed an increase of over £20, but that donations had diminished considerably. The treasurer drew special attention to the smallness of the amount received from collections in the churches and chapels in Reading. Subsequently the Rev. W. Neville, whilst admitting the difficulty of increasing the number of their special collections, said that at St. Mary's they intended to allocate at least £2 2s. a year from their sick and poor fund, and commended a similar course to other incumbents. The hon. secretary alluded to the presentation to the nursing staff, by the inhabitants of one of the poorest localities in the town, of a testimonial expressing the appreciation of a large number of women in that neighbourhood, accompanied by gifts for use in the nurses' sitting-room. It was also mentioned that owing to the increase of the work of the lady superintendent and her staff an increase of salary had been given to the nurses by the committee, who had under consideration the question of the engagement of an additional nurse. The number of patients nursed last year was 437, and the number of visits paid 12,474.

### DISTRICT NURSING IN CHELTENHAM.

At the annual meeting of the Cheltenham District Nursing Association, it was stated that a new feature in the accounts is the Fenn Memorial Fund, the income from which is to be employed in furthering the nurses' pension fund. We are glad that Miss Olga Hertz, who had been invited to give a special address at the meeting, expressed her surprise that the Victoria Home is not affiliated with the Queen's Jubilee Institute, and pointed out some of the advantages to be gained by affiliation. There is no doubt about the advantages, and we hope that they will be speedily recognised in the most practical manner. We should like to see all district nurses wearing the uniform of Queen's nurses, and in such a town as Cheltenham, there does not seem to be any reason why they should not do so.

### SHORT ITEMS.

It has been decided to form district nursing associations at Newburn, Northumberland, and Great Glen, Leicestershire.—Nurses Mary Thomas Buchanan, Ada H. E. Davies, Jane Barlow Didsbury, Janet Morrison, and Martha Musker of the Walton Workhouse Infirmary, Liverpool, Sister M. Sisson and Nurse R. W. Burrett, of Seacombe, Cheshire; Nurse Florence Anne Parkes, of Cheyne Walk, Chelsea; and Nurse Olivia Kemp, of the National Hospital, Bloomsbury, have passed the examination of the London Obstetrical Society and received its certificate.—There was a gathering of about 20 nurses at Marycourt, Bridgewater, the other day, to hear an address from Miss Eden on "Digestion and Diet."

### The Nursing Outlook.

"From magnanimity, all fear above;
From nobler recompense, above applause,
Which owes to man's short outlook all its charm."

### NURSES FOR WAR TIME.

At the time of the South African war, nurses willing to offer their services were invited to send their names for enrolment as an Army Nursing Reserve. It soon became apparent, that this system did not work satisfactorily, as it was beyond the power of those in authority, to ascertain accurately the true character and qualifications of many of the applicants, and their real fitness for work at the seat of war. The idea, however, was held by some people that an Army Nursing Reserve might be formed by keeping a list of all nurses wheresoever employed, who were willing to accept service with the army in any part of the world. This idea was felt to be impracticable by those who had an intimate knowledge of the circumstances of the nurses and the position of nursing in this country at the time. How, for example, could a nurse engaged in private nursing be expected to respond to a summons from the War Office, suddenly sprung upon her, when she was in charge of an acute case of illness, which might be approaching a critical stage? If she answered the summons and left her patient, her character as a private nurse would be seriously injured, should she resume the practice of her profession in the district from which she came. Yet on her return after foreign service, she would naturally feel drawn to the place where she was known and where she ought to find the best opening for work in consequence. Thus the idea of forming a Nursing Reserve by the means just explained was found to be impracticable and of little or no value to the country.

We have been interested to note that an attempt to establish by similar means a system in the United States for supplying nurses in time of war, or for national emergency, has in practice failed. The proposal was to invite nurses to send their names to the Surgeon-General's Office of the United States Army, without assuming any obligations to the Government beyond an expression of their willingness to offer their services when invited to do so, and to report to the Surgeon-General their addresses and the state of their health on January 1st and July 1st in each year. Although the scheme was approved by most of the superintendents of the principal training schools, who co-operated with the Surgeon-General to make it a success, the large and efficient body of eligible volunteer nurses which it was hoped to secure is still in the making. During the six months which have elapsed since the nurses of the United States were invited to send in their names to be placed on the list, although blank forms were sent to thousands of nurses to facilitate their making the application, only six names have been received. The American *Journal of Nursing* seems to attribute the non-success of this scheme to an absence of patriotism, or rather to habits of procrastination. We, however, are convinced, that the real cause is the difficulty which every sensible working woman must feel, for by placing her name on the Army List she might find it very difficult if not impossible for her, in certain circumstances, to discharge her duties to her patient in civil life.

No doubt it is desirable to prepare for war by organisation in times of peace. For then it is possible, as our contemporary insists, to avoid the enrolment of unsuitable women, who, under the pressure of war, might obtain entrance to the nurse corps, and by their unwomanly conduct bring discredit upon the entire nursing department. These undesirables might create such a scandal, that the splendid work of the many might be lost sight of and forgotten by the public. No one can question the wisdom of taking every precaution to prevent a risk of this kind, but the American suggestion, if it had attracted the nurses, would not have justified expectations or proved effective in practice, we are confident.

The only way to successfully establish a system whereby the country can be supplied in war time with an adequate number of capable nurses of the highest type is, for the War Office in this country, or the Surgeon-General in the United States to keep touch with the principal nurse-training schools throughout the country, and to arrange a plan whereby the managers of these institutions will be prepared to select and supply a given number of nurses when called upon to do so. All nurse-training schools, which are properly organised, not only have available a number of capable nurses, but they keep in touch with the post-graduate nurses whom they have trained, and are so in a position to secure the services of a number of the best type of women, as and when they may be needed by their country.

It is therefore for the War Office and the Surgeon-General to utilise the time of peace, by organising a definite system, whereby the co-operation of every reputable nurse training-school with the Army Medical Department will be secured. Under such a system, none but women of character would be sent out to the seat of war, and the prestige attaching to the selection, and the honour of upholding the reputation of each training-school, would prove in practice guarantees, that scandals would be avoided, honour increased, and reputation secured. Another and an important reason why the plan here suggested should prove efficient is, that the nurse training-schools would be able to arrange with the War Office, or the Surgeon-General, adequate terms for the equipment and pay of the nurses needed for army purposes, which would command the best services, and ensure the utmost satisfaction to all concerned.

### Christmas in the Fever Hospitals.

Any impression which may still prevail that Christmas in a hospital for infectious diseases is a miserable time will, we think, be effectually dissipated to-day. The descriptions we are able to give, written on the spot, of the actual modes in which the festival is celebrated, prove beyond doubt that the spirit prevailing in general and special hospitals, and in workhouse infirmaries, extends to the rapidly-increasing number of institutions which exist for the care of patients suffering from contagious maladies. It may have been supposed by persons who are unacquainted with the working of the most important isolation hospitals in the United Kingdom, that there were inseparable difficulties to Christmas rejoicing within the walls that shelter the victims of scarlet fever or diphtheria. They will get rid of this delusion when they have read the details of the clever and whole-hearted manner in which the members of the medical and nursing staffs, warmly, as a rule, supported by the governing authorities, address themselves to the task of making Christmas as bright as possible to the inmates; when they have seen the illustrations, which were taken last Christmas at the time the festivities were proceeding, of the visible signs of the efforts to provide the maximum of enjoyment to people of all ages who are necessarily, and most wisely, isolated from the rest of their kind while the world is keeping holiday. There are differences in the details; at some of the hospitals more is done, at others less. But there is one note in all—the note of a glad readiness on the part of those who serve to afford pleasure, and of keen appreciation on the part of those who are the recipients of the service. Not the least satisfactory feature in connection with the celebration of the festival in the infectious hospital is the fact that the patients are not forgotten by generous friends. It is true that the institutions are supported by the rates, but while we are convinced that the ratepayers who are well enough to spend their Christmas in their own homes, amongst the members of their own family, will not grudge the very small sum which is expended in order to brighten the period for those who are shut out from home and relatives, we are glad to know that the judicious liberality of the authorities is frequently supplemented by welcome gifts from outsiders. The remarkable improvements in the management as well as in the equipment of the principal isolation hospitals, the more ample provision for the patients, the higher qualifications of the doctors, the better training of the nurses, which are a guarantee of efficiency and comfort, would thus seem to quicken rather than to diminish the desire of the kind-hearted to render just the little help which is needed to give the final touch to enjoyment at the Christmas season.

### Grove Hospital, Tooting.

Christmas in a fever hospital. "How dreary!" methinks I hear someone say. Wait a moment, kind reader, and I will endeavour to alter your opinion. I have spent many years in hospitals, both general and fever, and I think as much healthy enjoyment is to be obtained in the latter as in the former, both for patients and staff. At the Grove Hospital, Tooting, Christmas begins some time beforehand; decorations are not tabooed as at a general hospital, because there are so few surgical dressings to consider. Paper flowers, fancy lamp shades, garlands and chains of various designs have to be made, and these afford keen enjoyment to the convalescent patient, who finds time somewhat tedious in winter. Even those who will not be in hospital for Christmas delight in helping, and it is no unusual thing to hear a patient say as he is leaving, "Well, good-bye old chap, I wish you were ready to come out, but you will enjoy Christmas here; I would like to come back for a few of the good things on that day;" and I remember last year one little one who was chained to bed for long weeks with paralysis confide in his Nurse that he didn't want to go home before Christmas. Christmas Day comes at last, and the little patients, who have been awake for a long time, are very excited about the contents of their well-filled stockings, hung up by Nurse on the previous night. In them are found toys, sweets, etc., which are supplied by the Committee, *Truth*, and other kind friends. At breakfast a Christmas card is handed to each patient, sent by the Chairman of the Hospital Committee. "How kind of him to think of me!" says one; "What a beauty!" is the remark of another. Nor is the staff forgotten; a similar token of remembrance is sent to each. Breakfast is quickly followed by the work of the day, for all has to be ready for the patients' dinner at 12 o'clock. Turkey, beef, fish, milk and plum-puddings, form the ménu. Dessert follows—grapes, nuts, oranges, apples, figs, dates, sweets, and crackers are provided for all who are fit to partake of them. Dinner over, willing hands, with heads adorned with caps and bonnets, speedily reduce the ward to its former state of tidiness. Then comes the fun. Nurses are allowed to visit other wards, provided they keep to the same disease as that which they are nursing. The convalescent patients, escorted by a nurse, are allowed the same privilege. In a male ward the patients gravely discuss the events of the day, while overhead hang the flags of all nations in various devices. Sombre evergreens show up the gay colours to perfection. No chains or lanterns are allowed in this ward; only lamp shades in our national colours throw a subdued light over all; while, on the table, plants and flowers make a perfect finish. In a children's ward merriment is at its height. To the strain of music emitted from a gramophone the little ones are dancing. A pretty sight! the girls in red dresses, white pinafores, and red hair-ribbons have for partners boys in bright blue suits. The colours in this ward are many and bright, but make a harmonious whole. Chains, lanterns in the shape of animals and birds, are fixed over the electric light; there being absolutely no risk of fire, Nurse conducts the proceedings with her mind at ease. Gay flowers, both real and in paper, with evergreens, and a prettily-trimmed Christmas tree finish this ward; and we leave it with a sigh. "How sweet, you say!" but what of these little ones after they go home?

Tramp! tramp! we hear as we leave the ward. What can it be! Going down stairs we come upon a strange cavalcade. A donkey bedecked as surely never was donkey bedecked before in ribbons, flowers, and a sun-bonnet; while a basket at its side seems to speak of more good things. Walking solemnly beside it is a well-known figure, Father Christmas. A little behind comes a wee, dancing fairy, and a small clown, who looks capable of playing any tricks, but who is walking along demurely at present. They enter a ward; shouts of laughter and clapping of hands greet their appearance. Father Christmas begins to speak.

### CHRISTMAS IN THE GROVE HOSPITAL, TOOTING—*Continued.*

"My children," says he; but the wig falls a little away, and reveals the well-known features of Dr. X—— ; and the rest of the speech is lost amidst much laughter. He gives away some of the contents of the basket and passes on.

Christmas for the staff begins after the patients are snugly tucked up in bed. In the nurses' home, which is remote from the wards, so that no sound of music or mirth can reach them, dinner is served at 8.30 P.M. for the charge nurses, the assistant nurses having had theirs in the middle of the day. At their dinners turkey and the usual Christmas fare is provided. After this is over the nurses who are not on duty in the wards are allowed to dance until midnight.

During Christmas week the Christmas trees which are provided for each of the children's scarlet fever and diphtheria wards are untrimmed, and the convalescent patients from the other wards are invited to take part, and great excitement prevails. The patients who are too ill to attend come in for a liberal share of the toys and other good things, and good-bye is said to Christmas once more until another year is past.

Grove Hospital, Tooting.

### Park Fever Hospital, Hither Green.

Christmas Day in an infectious hospital is, perhaps, more anticipated and more thoroughly appreciated than in any other institution owing, primarily, to the enforced isolation of the patients and staff, making life much more monotonous for both than in a general hospital, and secondly, because the patients are chiefly children, and we all know how very much the little ones enjoy Christmas time.

On Christmas Eve there is great excitement in the wards when all our little children, indeed some very big children, hang up their stockings, good Santa Claus, in the form of the night nurse, visiting them during the night, and filling each stocking full to overflowing with toys, bonbons, etc.

The editor of *Truth* is always exceedingly kind in sending us dolls and toys, and the managers of the Metropolitan Asylums Board are very liberal at Christmas time in allowing money to be spent for the patients, and I am sure if they could only see the happy faces and bright eyes of the children, they would feel more than repaid for their kind thoughts.

Some of the wards are very prettily decorated with flowers and evergreens, and in the evening fairy lamps and dainty coloured shades give an appearance of fairy-land.

An excellent Christmas dinner is provided for all the patients who are able to take it. Roast turkey, greens, potatoes, and plum pudding, followed by a liberal supply of fruit, such as grapes, apples, bananas, oranges, etc., to which I need hardly say they do full justice.

During the afternoon the convalescent patients sing songs and play games until tea-time comes when the tables are again prettily arranged with fruit and flowers, each nurse, trying to make her tea more attractive than the others.

Tea, consisting of the usual bread and butter and jam with additional cakes and biscuits, is thoroughly appreciated, especially in the male scarlet fever wards.

During the evening some members of the male staff give very great pleasure to the patients in the adult wards with violin solos, songs, recitations, and last, but not least, the gramaphone is always hailed with especial delight. Other wards are invaded by a small army of the domestic staff singing Christmas carols.

Christmas Day over, the staff are then considered, each section—charge nurses (or sisters), first and second assistant nurses, and the domestic staff, *i.e.* housemaids, wardmaids, the kitchen, laundry, and needle-room staff—being regaled on alternate days with a Christmas dinner, and the tables prettily decorated with fruit, flowers, and bonbons.

The officers assist in waiting at table, and at the domestic staff dinner, many of the nurses volunteer to help, and not only wait upon the maids, but also clear the tables and wash up the dishes.

During the week the nurses are given a dance, when the doctors join them, and although there are necessarily so few men, everyone enjoys it.

Park Hospital, Hither Green.

Bedtime comes only too soon, and the little ones, hugging their toys, are very soon in the land of dreams.

The whole of the nursing staff are on duty on Christmas Day—passes, except for early morning church, not being granted. This is done to enable the nurses to devote more time to the amusement of convalescent patients.

The dance takes place in the nurses' home, which is situated some distance from the wards, the assistant nurses' dining-room being used for dancing, and, with a good floor and good music—a pianist is engaged for the occasion—the evening is a great success, and every one is sorry when the time comes to say "Good-night."

### City Hospital, Grafton Street, Liverpool.

On account of the isolation naturally associated with fever hospitals, it might be supposed that in such institutions the usual Christmas merry-making would be rather up-hill work. Such, however, is not the case, at least, so far as Grafton Street City Hospital, Liverpool, is concerned. Necessarily the staff are, to a slight extent, deprived of outside help, but this seems merely to have the effect of stimulating their individual efforts, and the entertainments projected and carried out are highly creditable.

For days before everyone is busily engaged in decorating the wards—holly, mistletoe, and artificial flowers made by the patients being the pervading features. Much of the nurses' spare time is spent in rehearsing songs and choruses, in drilling such of the juveniles as are able to take part in

### CHRISTMAS IN THE CITY HOSPITAL, GRAFTON STREET, LIVERPOOL—*Continued.*

the performances, and in the designing and putting together of fancy dresses.

On Christmas Eve the children sing carols; after which is much eager anticipation and excitement over the hanging up of numerous small stockings. Santa Claus (for the time being disguised in a cap and apron, and minus a beard) has no fear of infection, and empties his sack of toys and sweetmeats with a liberal and impartial hand. But not only among the children, for the grown-ups, were they overlooked, would be much disappointed, and there is much fun and laughter when the time comes in comparing the various gifts.

In the morning—very early in the morning—sundry of the small fry are awake, bent on making the most of the day that comes but once a year. Besides presents, there is for everyone in the building a letter, sent by an outside lady friend who is interested in the hospital.

The usual rounds by matron and doctor being over, the next proceeding is Christmas dinner, the importance of which has been duly recognised by cook, and the generous fare provided is suitable to the occasion. Games and a general play-time follow, till comes the great event of the day—the Christmas tree, which is to be despoiled of the many toys and other articles with which it is laden. This is a duty which falls to the doctor, and as he reads out the name with which each present is ticketed, the recipient comes forward to accept it. There is something for everybody—matron, doctor, nurses, patients, and servants.

The Christmas tree is not kept as a surprise; indeed, the children themselves deck it, the one reservation being in the case of their own gifts, which are hung while they are on other things intent. The rest of the day is spent in amusements of various kinds; tea is served at long tables in the ward; and soon there are signs that a long and very merry day has come to a close.

The entertainments which take place during the week following are of the "Variety" order. Songs, choruses, and a little sketch or one-act farce, are given by nurses, patients, and doctor, most ably seconded by a troupe of amateur Pierrots, whose humorous recitations, amusing speeches, and acting, are a prominent feature of the evening. Everything has been carefully practised, and if some of the youngsters are a little nervous on making their début in public, their appearance is none the less delightful. Last year a special stage was procured for the hospital; effective electric footlights were fitted up by the engineer, and the result amply repaid all the trouble which had been taken to produce it. The audience was most appreciative, and those of the acute cases who passed muster at the doctor's hands, had their beds wheeled into the convalescent ward, so as not to miss any of the fun—a kindness which, to judge from the eager delight with which they watched the proceedings was not thrown away.

Everything, however, must have an end. At last the signal for breaking up is given; all join hands in singing "Auld Lang Syne," and the whole winds up with a hearty rendering of "God Save the King!"

City Hospital, Grafton Street, Liverpool.

### City Hospital, Lodge Road, Birmingham.

How Christmas Day and the days following are spent by the inmates of a fever hospital is a subject about which information is usually gleaned through the medium of the press. The infectious nature of the cases and the stringent regulations as to visiting by patients' friends and others combine to make such hospitals really "isolation" hospitals, and the meaning of this term has a double significance at festive seasons.

In this hospital, containing over 300 beds, where the majority of patients are suffering from scarlet fever, and consequently at an age when Santa Claus is not a mythical person, the labour of decorating the wards and providing entertainments and other good things, is really one of joy. The pleasure of seeing how the little ones, separated at such a time from their homes and parents, forget their troubles and enjoy, as only children can, the toys, dishes, and delights provided, is ample reward to the nurses.

For some weeks before the expectant day a superficial observer would probably not notice anything unusual in the hospital, but a more intimate knowledge of its routine would reveal a certain bustle and stir among the staff which portends one thing only. Just a couple of days or so only before Christmas do the labours of these weeks spring into evidence. The wards become suddenly transformed from their usual every-day appearance into what can only be described as "bowers of beauty." Appropriate mottoes, touches of evergreens, Christmas trees, the draping of screens, the mellowing of light by coloured shades—these among many tend to give the wards a seasonable appearance. Christmas Day is early heralded. Somewhere in the early hours all the members of the nursing staff meet, and each one provided with a Chinese lantern on a staff, form into marching order. They then slowly wend their way through every ward and the administration buildings, singing Christmas hymns and carols. The effect of such "pilgrims of the night" is very striking, and an appropriate beginning to the day. The patients, including the few adults, find on awakening that Santa Claus has not been remiss. Each one has a well-filled stocking hanging up at the foot of the bed or cot. Breakfasts are served as usual, and the wards visited by the medical officers and matron. At this time, also, the Chairman of the Birmingham Health Committee, pays the hospital a visit — a pleasing duty which he has not missed for many years. Inside hospitals, as elsewhere, the Christmas dinner is all-important. The fare provided is sumptuous—goose, turkey, chicken, plum-pudding, and fruit. The patients' dinners are begun at 12 o'clock and nearly all have medical permission to partake freely. This they do, and what with sweets and bon-bons the meal lasts about an hour. At two o'clock all the maids and porters sit down to a long table prepared in the hospital kitchen, and an hour later the whole nursing staff dine together in another large sitting-room. The fare provided here, as elsewhere, is seasonable, and many of the patients and staff have their futures sealed by finding such things as thimbles, rings, etc., in the pudding. Tea in the wards is then served, cakes and other dainties forming part of the fare. Having thus well-satisfied the inner man, the patients gather together in the recreation-ward, where two hours are

City Hospital, Birmingham.

CHRISTMAS IN THE CITY HOSPITAL, BIRMINGHAM—*Continued.*

pleasantly spent, First of all a magic lantern entertainment is given by the medical superintendent. Following this a very pretty and humorous sketch is performed by eight of the night nurses, captained by the night superintendent. As evening approaches the little patients begin to succumb to nature's call, and are soon back in their respective wards, sleeping the sleep of the " weary but contented." The medical superintendent, matron, and sisters dine together later, and, as a wind up for the day, those nurses who can be spared from the wards repair to the recreation ward where games and dancing are indulged in till midnight. The maids also are allowed a similar privilege in their large sitting-room. The "stripping" of the Christmas trees takes place on the days following, and at each function games are instituted for the convalescent patients. The decorations remain fresh and green and are kept up till early in the new year.

### Borough Isolation Hospital, Leicester.

CHRISTMAS DAY is heralded in by sweet music, for the Temperance Hall Brass Band has come, according to custom, to play at each ward entrance the old familiar carols. The little ones, roused from their sleep, remember that the long-looked-for day has come, and wonder whether Santa Claus has indeed visited them.

Borough Isolation Hospital, Leicester.

Wonderful have been their anticipations, and great is their delight at finding them realised! That he has really come down the chimney whilst they were sleeping soundly there seems now no room to doubt. For oh! the wonderful happenings of the night! Such lovely toys, as if by magic, have sprung into existence. The tree, last night so bare, is now transformed into a vision of loveliness. Stockings which were hung over-night with such anxious care, lest they be overlooked, are found to be filled with oranges, apples, goodies, and bon-bons, with perhaps a dolly or some other long and eagerly wished-for toy.

All these treasures provide ample occupation until dinner time, but do not prevent justice being done to the roast fowl, plum pudding, and other good things provided for that meal.

After dinner comes the great event of the day. Father Christmas, clad in a bright red snow-trimmed gown, his long white beard glistening with frost, accompanied by three fairies, bearing between them a capacious basket brimful and running over with presents for all, makes his way to all the wards in turn. Books, toys, fruit, chocolates and tobacco are amongst the gifts which he bestows with a few words of advice culled from an experience of "nineteen hundred odd years." The juveniles—a trifle awed by his venerable aspect, yet happy and pleased and excited, will think and talk of little else for days to come.

Then a sumptuous tea is followed by some games—blind man's buff and hunt the slipper being great favourites with the convalescents, and the great day is over. Little heads begin to nod and eyelids to droop, for Christmas Day, as well as being the happiest, is the busiest of all those spent in the fever hospital, and the memory of it will outlive that of sickness and separation from home and friends.

### Leeds Fever Hospital.

A STRIKING feature of Christmas in hospital is surely the fact that one and all conspire to make it in all truth for the patients "A Happy Christmas." The old-time custom of carol-singing commences our Christmas, when the sisters
The festivities begin in real earnest later when the patients gather round the flower bedecked tables to partake of Christmas fare. Proud and happy are those in bed who are voted by the doctor as being well enough to share
and nurses, starting at the doctors' and matron's quarters on Christmas Eve, visit each pavilion singing carols and Christmas hymns.

"Santa Claus," who visits during the night, is responsible for much happy excitement in the early hours of the morning when the children wake to find, in stocking or parcel, gifts of toys, sweets, etc., dear to the hearts of the little ones. The day nurses are greeted on all sides by requests to "come and look at this, nurse; I got it in my stocking." A pleasing incident in the male ward is Sister's reception on Christmas morning. The prettily decorated ward with its rows of fresh white beds, the bright faces of the sick patients, the assembled convalescents who are grouped round the piano, the hearty voicing of "A Happy Christmas, Sister" with which, by one accord, they greet her, and afterwards the still more hearty singing of a Christmas hymn, all make an impression not easily forgotten.
it, and even for the others some little change of diet is made. Beer is provided for the men, and cigars and cigarettes, are presented by a kind friend. An excellent dinner of goose or turkey, plum-pudding, mince-pies, etc., has full justice done to it. A pleasant feature of our Christmas Day is that the doctor and matron, with their assistants, preside at the patients' dinner.

A Ward in the City Hospital, Leeds.

Afterwards we have music and games, and those patients who are able to do so assemble in the children's ward, where they are entertained by the young people themselves.

The little girls, in white pinafores and bright hair

City Hospital, Leeds.

**CHRISTMAS IN THE CITY HOSPITAL, LEEDS**—*Continued.*

ribbons—the latter the special gift of the doctor—and the boys, also at their best, look charming.

Some of the children, trained by the sister and nurses, give a pleasing little entertainment, consisting of songs, an illustrated rendering of several nursery rhymes, also a pretty tambourine drill. The clever handling of the gaily be-ribboned tambourines affords much pleasure to the audience.

The children's ward, decorated to represent "Fairyland," looks delightful, and their dinner table has for a centre-piece a "May-pole," each ribbon being held by a doll, daintily attired as a fairy.

The "Christmas Tree" is, of course, a real delight to the children.

Then comes tea and Christmas cake, after which commence preparations for the evening's concert, which is held in the male ward, where a platform is erected.

At six o'clock the "Minstrel Troupe" appear, delighting their audience, which, by the way, includes the older patients from all other wards, by the bright rendering of some favourite "coon" songs, choruses, etc.

The "Minstrels," who belong to the nursing staff, are excellent amateurs, and their performance is thoroughly enjoyed by all. All too soon comes the singing of the National Anthem, and the fall of the curtain for the last time.

The decorations in each pavilion are exceedingly pretty, the walls with their delicate colouring admirably lending themselves to the trailing greenery and bright mottoes. The dinner tables, with their flowers and smilax, bright crackers, and sweets, are a pleasant sight, and these, with the well-laden Christmas Trees, are much enjoyed by the patients, who, at the close of the day, always agree that they have had "a right good time," and would never again be afraid to spend Christmas Day in hospital.

### City Hospital, Newcastle-upon-Tyne.

THE Christmas festivities begin on Christmas Eve. Last year there was an entertainment in Pavilion A. The recitations and songs were admirably rendered by the children in costume. Where the dresses came from puzzled the audience until it was explained that condemned sheets had been saved for a long time and laid aside for this purpose. The ward was prettily decorated and well arranged for the concert, and the repeated encores showed the appreciation of the audience. It is the rule to have the large pavilions in use decorated and each has its Christmas Tree. As soon as the decorations begins no one from one pavilion is allowed to see what is being done in the others, and in consequence each ward has a character of its own. Not only do the inmates of the wards work long and late to make pretty things, but old patients also send gifts in memory of their stay here.

City Hospital, Newcastle-on-Tyne.

Late on Christmas Eve a body of carol singers go round the grounds singing very sweetly so that, though we are shut out from the rest of the world by a high wall, we have our Christmas as complete as possible.

Early on Christmas morning, before the sun is well up, Santa Claus starts on his rounds with his sack full of toys. The children hung up their stockings for him but some of them are surprised and even a little frightened when he really appears. They soon get over their fright, though, and are delighted to see at last a real live Father Christmas.

The morning is taken up with the new toys sent in by kind friends and later on with preparations for the Christmas dinner. A band of willing helpers come out to escort the dinners from the kitchen and soon all are busy with their meal. The patients in bed are, when possible, brought into the wards where the dinners are going on so that they may see the pretty sight though they cannot in all cases share in the good things.

The afternoon is taken up with games and at the end of a long and exciting day the children go off tired to bed. The older patients work hard to help the nurses with the decorations and do their best to make the day a happy one.

After the patients' dinner is over, the doctors and the matron have their Christmas dinner with the nursing staff. Everyone is remembered, and later in the afternoon the matron holds a reception at which all the nurses are present.

### Borough Sanatorium, St. Helen's.

Christmas Day in the scarlet fever pavilions is ushered in by the blowing of trumpets, whistling, and delighted shrieks from the children when it is discovered at 6 A.M. that "Santa Claus" has been round the various cots and deposited gifts in each tiny stocking.

Borough Sanatorium, St. Helen's.

All the patients, with the exception of the acute cases, are taken to one of the large pavilions for dinner, where full justice is done to the Christmas fare provided, consisting of round of beef, plum puddings, and dessert. The tables are prettily decorated with chrysanthemums, plants, and bon-bons. Afterwards the children are engaged in games of all kinds and dancing.

The nurses are indefatigable in their endeavours to make Christmas a merry time for their charges. Happy but tired children troop off to bed at six o'clock to dream of the Christmas tree to come, when another delightful afternoon is spent by all, and each individual desire, in the shape of a toy, is supplied by Father Christmas, whose approach is greeted by loud laughter from the boys, though two or three babies announce their consciousness of his presence in not quite so cheerful a manner. Christmas is spent in a more sober manner in the enteric wards; but even the worst case amongst the patients rouses himself sufficiently to take an interest in the contents of his "Santa Claus" parcel; indeed, the night nurse has often little humorous anecdotes to relate about the same parcels. One man actually refused to take his pipe out of his mouth for fear it should disappear; another thought that he would go to sleep again and see if anything else would turn up.

Dinner is served to the convalescents in an empty ward,

BOROUGH SANATORIUM, ST. HELEN'S—*Continued.*

for fear of upsetting those who unfortunately feel well enough to eat but are not allowed to do so.

The tables here also are charmingly arranged with flowers, smilax, and ivy; all do ample justice to roast turkey and dainty light puddings. During the afternoon carols are sung by the nurses, in which some of the more musical of the patients join. A rough collier, on being asked how he enjoyed his Christmas, replied in broad Lancashire: "Reet enough, but there's nought of it."

What these patients lack in the way of Christmas fare and amusement is made up to them in decorations. The men and women who are able to do so both help and give advice as to the manner and method of arranging and hanging the festoons, and much credit is due to them. The doctors are good enough to tell them it is like fairyland; the visitors much gratify their sick relations by saying that their wards are the prettiest.

The decorations in all the pavilions are tastefully carried out, festoons of coloured paper, drapings of prettily-coloured art muslin, chains of evergreens, electric light shades of crinkled paper caught up by butterflies, lanterns, and flags, lend an air of gaiety to the bright, handsome wards.

The entrance comes in for special attention, and in some the style is both gorgeous and original; in the largest pavilion the entrance is further embellished by a huge "Christmas Tree" decorated most beautifully, and lighted up by electric lights, laden with presents for every one, including doctors, nurses, and servants. The distributions cause a great deal of merriment, and the evening winds up with a musical entertainment and dancing.

### Ruchill Fever Hospital, Glasgow.

It must be very apparent to outsiders that Christmas in an isolation hospital is of necessity a much quieter season than in a hospital or an infirmary for general diseases. The strictness of the rules with regard to infection, the care with which the welfare of the outside world is considered, prevents the rejoicings, from being anything but local. Outside visitors are not allowed, so parents, brothers, and sisters must be content with making inquiries at the gate; only the clergy are admitted to the ward without question. Nevertheless Christmas is not forgotten, and there is no class of people so able and willing to make the season enjoyable as the hospital nurse on Christmas Day morning at Ruchill Hospital. There is something everywhere to remind one of the season, from the few branches of holly over the clock, to the most elaborate designs in mottoes and festoons. All this is the work of the sisters and nurses, whose willing hands work the mottoes, and even gather from hedgerow and garden the holly, ivy, and laurel. The day is devoted to entertaining the patients; there is a tea party in each ward, presided over by the sister in charge. Scones, cakes, and fruit are provided from the capacious cupboards of the steward. In a few of the wards a little concert is arranged amongst the convalescents. The nurses' Christmas dinner is at two o'clock in the afternoon, and is presided over by the chairman of the health committee. It consists of the usual

Ruchill Fever Hospital, Glasgow.

Yuletide fare. The bairns are kindly thought of by the Glasgow people who, when ordering toys for their little ones at home, do not forget the little sufferers during their compulsory stay in the city hospitals, but send a parcel for the sick as well.

When these are distributed, it would amply repay the donors to see the joy with which the gifts are received. New Year's Day is brought in with a little dance confined to the nursing and medical staff, the latter numbering five, yet in spite of the scarcity of the sterner sex, the nurses manage excellently, and a most enjoyable evening is spent. On the stroke of twelve o'clock a rush is made for particular friends. Hand-shaking and good wishes to a perfect babel of chattering goes on for the next ten minutes, when the fun of the night closes with "Auld Lang Syne," and "God Save the King."

On the first day of the year all patients who are well on in convalescence have for dinner roast beef, vegetables, and the much-loved plum pudding. Those in the medium stages have roast chicken or fish and light puddings, but a goodly number can have nothing to remind them of the festive season, being too ill to go beyond the usual diet in the early stages of fever. While all this is transpiring, the domestic staff is not forgotten. They have their dinner on New Year's Day, which is similar to the nurses'. Also a little dance held in the ironing-room of the laundry a week later, where the nurses again assist in the entertaining and providing of music. Of course all these rejoicings mean a deal of extra work, but the toil is given by doctors, sisters, and nurses with willing hearts. Their only reward is the smile that greets them as they go about their respective duties in the wards. By the world unseen and by the world forgotten, they desire no man's praise, but proceed with their duties as those who have found a noble vocation which brings its own reward, content, when prosaic life again comes around, to know that they have cheered the hearts of the sufferers.

### House of Recovery and Fever Hospital, Dublin.

CHRISTMAS in a fever hospital is necessarily much quieter than in a general one. There are no "outside" visitors giving "teas," and bringing their friends to help to entertain the poor sick people. There cannot even be one coming back from church there is breakfast and a hurried look at presents and cards before going on duty.

The wards are prettily but not heavily decorated with holly and ivy on the gas-brackets and centre pillars, also on

House of Recovery and Fever Hospital, Dublin.

"joint" gathering inside the hospital, as each disease has to keep to its own house with its own nurses for the time being.

We do not begin with carol-singing on Christmas Day, but every nurse goes out to early Mass or service. On the landings. Plenty of nice plants and flowers make the tables look bright.

In the enteric wards things are very quiet. A good many bad cases are generally in, and under these circumstances

FEVER HOSPITAL, DUBLIN—*Continued.*

the only festivity is a small Christmas-tree on the ward table decorated with toys.

In the diphtheria wards it is usually a little brighter. Here, too, is a small Christmas-tree loaded with toys, oranges and sweets. The diet also is not so strict. The erysipelas, measles, and whooping-cough wards are much the same.

In the scarlatina wards things are quite different. From early morning the noise of "musical" instruments is heard, and great excitement prevails amongst the many convalescent children. A large Christmas-tree is set up in one ward, and on the afternoon of St. Stephen's Day the toys are distributed. One of the resident medical officers impersonates an old Irish witch and hands the toys round. Afterwards another of the residents plays some selections on the violin, which are greatly appreciated, and a very pleasant afternoon is brought to a close. The staff has all the usual turkey and plum pudding fare on Christmas Day. On Monday, Tuesday, and Wednesday of the following week, the sisters, assistant nurses, and probationers have each a long evening off duty, when they amuse themselves with dancing, music, and fortune-telling, and a special supper, with all good things is provided each night. One night the sisters are sent to the pantomime which they enjoy more than anything.

Christmas Tree in Small Scarlet Fever Pavilion, Allt-yr-yn Hospital, Newport.

### Allt-yr-yn Hospital, Newport, Mon.

FOR several days before Christmas Eve an air of suppressed excitement pevades the atmosphere of the hospital, penetrating even the acute ward, and extending to the typhoid and diphtheria pavilions. There are mysterious shopping expeditions and whispered conclaves among the nurses, and matron is asked not to look into this and the other cupboards, matron's strong point being tidy cupboards. The decorations usually do the nurses great credit, and only the finishing touches remain for next day, such as arranging cut flowers and new plants to the best advantage. Stockings are hung up; in most cases two hang from each bed, and there are instances of a small pair of trousers being substituted by the youthful owner, who takes the precaution to tie the legs up. Discussions are numerous and varied as to Santa Claus' expected visit: "Go on, you silly, how can he come down the chimbley when there ain't no chimbley there, them stoves don't need no chimbley?" but, discussions over, every youngster retires fully determined to keep one eye at least open for Santa Claus. The adults smile indulgently and say nothing. In spite of the intention to keep awake, the wards soon become very quiet, except for the sound of sleep in different keys. Midnight comes, and so does Santa Claus. He does not visit this

Verandah, Diphtheria Ward, Allt-yr-yn Hospital, Newport.

hospital as he is usually depicted—a jovial, rosy-faced old man with white beard, cosily clad in scarlet garments trimmed with white fur; our Santa Claus is a strangely muffled figure bent down by the weight of a piled-up tray of white paper parcels of different sizes, instead of the bags and hampers of the orthodox Santa Claus. He is closely attended by a satellite also bearing a tray, and the attendant has a strong resemblance to the night sister. Perhaps they are relations. However none of the patients are awake to see or criticise appearances. Some of the parcels are much too big to get into the stockings. Santa discriminates fairly well as to which patient requires warm under-garments; to those who are in possession of all such necessaries he brings gifts of initialled silk handkerchiefs, silk ties, and similar articles. Then two oranges, a box of chocolates and odd little things are dropped into each stocking, and Santa bids adieu to the night nurses after a visit of a couple of hours' duration or so.

Oh! the waking up in the morning! the excitement of Christmas greetings! and the fumbling with string to find out the contents of the parcels! Breakfast over and oranges disposed of, lunch comes next, and matron's visit. Santa's presents are displayed and admired. Then the nurses again vie with each other in the laying out of tables and the arranging of dessert. The medical superintendent and matron carve the turkeys and hand round vegetables; the turkeys are the annual gift of a kind gentleman who does not like his name mentioned; the green vegetables are from the kitchen garden. Plum pudding follows; dessert consists of apples, oranges, grapes, bananas, raisins and almonds, nuts in moderation, bon-bons galore,; lemonade is the beverage drunk. Dessert lasts until nearly tea time. Matron, sisters, and nurses all have tea in the wards on Christmas Day; it is jollier for the patients, and allows the domestic staff to go on with preparations for their dinner party, which lasts from 6 o'clock until 11 P.M. The patients from the acute wards who are able to attend are the guests of the convalescents in the afternoon. After an extra nice tea of cakes and biscuits, games are played and singing indulged in until supper time, then bed, and so finishes Christmas Day amid expressions of satisfaction from nurses and patients alike. The next function is the Christmas Tree, when a number of friends who defy germs come to assist in stripping and distributing the toys. The typhoid and diphtheria patients have their own little trees, but *the* tree is a lordly fellow rich in toys which have been subscribed for by numerous well-wishers and old patients. The tree has been the yearly gift of a nobleman in the district for several years. After the festivities of the patients comes the nurses' dinner, when a few guests are invited to dine and a few more come in after dinner for games, music, and a little informal dance. This usually occurs on Old Year's Eve, and after listening to the muffled peal and the joyful ringing in of the New Year the guests depart, as early hours have still to be observed the following morning, and so passes the Yuletide at this little hospital.

### Guy's Hospital.

ON Tuesday evening a concert will be held in the Court Room, Guy's Hospital, under the direction of Mr. Henry Taylor. The programme, which is of a varied and interesting character, will include solos by Sister Lazarus, Nurses Dickenson and St. John. Nurse Woodward will contribute a violin solo, and Nurse Margaret Taylor will act as accompanist.

### The New Norland Nurseries.

THE Norland Institute, for the training of gentlewomen as children's nurses on Froebelian principles, was founded 12 years ago by Mrs. Walter Ward, and is under the entire management of a principal and her assistants who hold certificates either of the joint board of the National Froebel Union, or diplomas from some technical training school of good standing. The principal, Miss Sharman, holds a first-class higher certificate of the Froebel Society.

Great improvements have recently been made in the scheme of training. It now extends over a period of one year, and includes three months' experience in a children's hospital. The domestic and educational part of the training is obtained at the Institute, 10 Pembridge Square, W.; while the last 10 weeks of the course are now spent in the new practising nurseries at 7 Pembridge Square. The certificate of the Norland Institute cannot, however, be obtained until the probationer has completed six months' work in a family.

Last Saturday afternoon the new nurseries were on view, and a good many people, chiefly ladies, inspected them. Mrs. Ward gave two explanatory addresses, and each nursery had a visitors' tea party. There are six model day and night nurseries, and about eighteen children can be received. At present there are seven little inmates, four of them being babies. Each nursery looks most inviting, and one called "Forget-me-not" is really beautiful. Another named "Speedwell" is also very attractive. Two nurses are allotted to each nursery, the senior nurse sleeping in the night nursery. The nurseries are under the direction and supervision of two experienced Norland nurses, who, as matron and head nurse share the entire responsibility; and the present head nurse has been at the Hospital for Sick Children, Great Ormond Street. The arrangements of the nurses' quarters and also of the kitchen are admirable, but the ventilation does not appear to be adequate. Each day nursery leads into a night nursery, and a special feature in the day nursery is a food cupboard, or safe, with a perforated metal back, in an outside wall. Gas stoves are fixed in the night nurseries.

Children from one month old up to seven or eight years of age can be received in the nurseries, which are intended for "the children of Indian officers and others on foreign service; widows, widowers, members of the theatrical profession, guardians, trustees, missionaries, colonials, or parents desiring a temporary, safe and happy home for their little ones while they travel." Infants and little children are taken by the week, month, or year. The nurseries do not depend entirely on the fees paid by the children, as they form a practising school for the Norland Institute nurses. The charge for infants from one month, and until the period of teething and bottle-feeding is over, is from 15s. to £2 2s. weekly, and for children beyond infancy from £38 to £100 a year. The children are fed on what seems to suit them best, not according to the fees paid. All children receive the same care and attention, but of course those who pay best get the best rooms and other advantages. There are no extras of any kind, except medical attendance. The home takes the risk of whooping-cough, measles, and chicken-pox, if contracted while in the nurseries; but in the case of any serious, infectious, or contagious disease, a child may be sent to a hospital unless the extra expenses of nursing and isolating such a case be guaranteed by the person responsible for the maintenance of the child. Relatives of the children can visit their own nurseries practically at any time, and the matron or head nurse can always be seen.

### The Japanese Army Nurses.

MISS ETHEL McCAUL'S EXPERIENCES.
BY A CORRESPONDENT.

The experiences of Miss Ethel McCaul have just been published in book-form. A representative of The Hospital *Nursing Section*, who had an interview with the author of "Under the Care of the Japanese War Office," at Welbeck House last week, elicited from her some interesting information respecting the Japanese Army nurses.

Having the sanction of Queen Alexandra, and the permission of the Japanese Government, Miss McCaul left England for Japan last March under exceptionally auspicious circumstances. Every facility for seeing the work of the Japanese military nurses was given her, and when she proceeded to the front, she was provided by the Japanese Government with quite a retinue, deputed to accompany her during her sojourn in Japan, including a business manager, whose duty it was to keep the War Office informed of her movements.

"It was so like the Japanese Government to do that," said Miss McCaul. "The first thing they did for me was to provide me with a Japanese lady, Madame Kuroda. She spoke English perfectly, accompanied us everywhere, and proved

Miss Ethel McCaul and the Japanese Nurses.

to be a delightful and invaluable companion. Miss St. Aubyn, as you know, went out with me from England, and was with me all the time. I was more than pleased with nearly everything I saw in Japan, and their Red Cross Society is a wonderful organisation."

"In time of war," continued Miss McCaul, "orderlies are sent to the front, and women replace them in the military base or reserve hospitals. The nurses are all fully trained—for three years at least. Their uniforms consist of white cotton dresses, with a very broad, stiff, white waistband, white socks and straw sandals, and a white mob-shaped cap with a red cross in the front. They do not wear aprons, but in the operating-theatres they put on white overalls with short sleeves. I can show you the cap."

Miss McCaul having produced the quaint cap, which is large and severe-looking and entirely covers the front of the hair, proceeded:

"The Japanese nursing is really excellent. You see their one idea is patriotism and entire obedience. The discipline is splendid. These women, too, with their marvellous self-control, quick intelligence, and general activity, seem the very people to wait upon the sick; and they are so clean and dainty in all their ways, and have such small, delicate hands. All Japanese Cross nurses are army nurses."

"Then about the hospital army ships?" I asked.

"We were on one of the Red Cross hospital ships, the *Hakuai Maru*, for several days on the way to Antong, and I will show you a photograph of us taken with the nurses on deck. You will see that the uniforms are rather different here. They are of blue serge, made in the same style as the white, but not nearly so becoming. It is their outdoor uniform though, and is not worn when attending patients. The Japanese lady in European dress is Madame Kuroda."

"You saw the work of the Red Cross Society in the field?"

"Yes, at Feng-hwang-cheng, beyond Antong. We also visited Matsuyama, where the Russian prisoners were detained. I should like to add that the Empress, who gave me the highest order of the Red Cross Society, takes a great interest in the hospitals and visits them personally."

Miss McCaul then told me something about private nursing in Japan. It seems that before a woman can start nursing on her own account, she must report herself to the town authorities in the district in which she intends to work. She has, of course, to state her qualifications. The authorities then ratify her papers, and if they are all that can be desired, she is granted a license to nurse in that particular district.

"It is an excellent arrangement," said Miss McCaul. "I only wish private nursing in England could be better regulated. Quite apart from the question of State registration, why should it not be illegal for any untrained woman to wear nurses' uniform?"

"What did you think of the American nurses who went to Japan?"

"The idea, of course, was absurd. They were not in the least wanted. The Japanese behaved very nicely and courteously to them, giving them a grand reception, but they never intended to let them do much."

### The Value of the Croydon Certificate Restored.

After years of persistent striving the Croydon Board of Guardians have at length given way to the desire of the nursing staff of the infirmary, and have agreed to restore the matron's signature to probationers' certificates. The signature of the matron was removed by order of the Board in September 1900, and from that time up to the present the probationers have not ceased to agitate.

About a month ago the Infirmary Committee received a petition from the staff again asking for the concession. The committee reported against granting it, but the Board referred the recommendation back to the committee by a substantial majority.

At the meeting of the Board on Tuesday, the infirmary committee reported that they had further considered their report of the 1st inst. on the subject of probationer nurses, as desired by the Board, and they decided to advise the Board to take steps to restore the matron's signature to the probationer nurses' training certificates. Before this suggestion can be carried out, the following resolution of the Board of September 18th, 1900, must be rescinded by resolution, after formal notice, as per the Standing Orders, viz., "That the matron's signature to the training certificates of probationer nurses be dispensed with, and that the existing form of certificate be reprinted, omitting the place for the signature of the matron thereon."

The Board adopted the report, and Dr. W. B. Addison at once gave formal notice that at the next meeting he should move to rescind the resolution of 1900.

### Queen Charlotte's Hospital.

ALTHOUGH it has been for some time known in the hospital that Miss McCord was relinquishing the post of matron, it was only the week before last that the vacancy was publicly advertised in our columns. Trained at Crumpsall Infirmary, Miss McCord subsequently held a post at Carlow, which she relinquished in order to take up the work at Queen Charlotte's Hospital. During her ten years' residence the regulations for the training of midwives and monthly nurses have been greatly improved; the term of training has been increased for midwives to five months, and for monthly nurses to four, while in other respects the rules have been so reformed that very trifling alterations were required to enable the hospital to comply with the standard set up by the Midwives Board. It was largely through Miss McCord's energy and foresight that the new home for the nurses and pupils was erected in Marylebone Road, to which, owing to the increasing work in the wards, an extension is now in contemplation. During her term of office the hospital has been federated to the Royal National Pension Fund for Nurses. Miss McCord leaves England next week in order to take up an important post in Italy.

### Everybody's Opinion.

### LADY DUFFERIN'S FUND AND THE SUPPLY OF NURSES.

MISS E. A. MANNING writes: Will you allow me to state, in reference to a reply to a query on page 102 in the *Nursing Section* of your issue for November 12, that nurses are not included in the scope of the National Association for Supplying Female Medical Aid to the Women of India, founded by Lady Dufferin. As I have received several inquiries from nurses since my name was given, I shall be obliged if you will insert this in order to prevent disappointment. I may add that experienced nurses wishing to go to India can sometimes hear of appointments through Mrs. Sheppard, 10 Chester Place, Regent's Park, N.W., under the Up-Country Nursing Association.

### ANOTHER WARNING FROM SOUTH AFRICA.

"SISTER FRANCIS" writes from Johannesburg: I would like to send a note of warning to my fellow nurses in England, who may be contemplating coming to South Africa. I can only repeat "Punch's" advice to those about to marry, "Don't," especially with regard to Johannesburg; it is indeed a place to be avoided just now as the wave of depression is so great that the nurses who are here find it very hard work to make a living. For instance, a Queen Charlotte's nurse holding excellent certificates was quite recently only too glad to take a position as a children's nurse at a small salary, in which position she is at the present day. The fees sound tempting, but in England it is impossible to realise what it costs to live here. It is almost impossible to do so under £3 a week, when a nurse is not at work, as £4 to £5 a month has to be paid for rooms and food is also very expensive. Eggs, for example, are 4s. to 5s. a dozen, and everything is on the same extravagant scale. Nurses have always realised that if they only possess certificates for medical and surgical work they often have many weeks of idleness during the year. Maternity nurses are generally supposed to be kept better employed. Now here a great many maternity nurses have only done about two weeks' work out of each month for some considerable time past. The fees being high a number of people can only afford to keep the nurse for a fortnight, in fact it is very rarely that she stays for three weeks, and more often than not the mothers manage without a nurse at all. The medical profession agree, that the health of the town has improved, as it always does when the sanitary arrangements get properly looked after. Just one word more. If a nurse decides to come here, she should have at least £100 to her credit after she has paid all her travelling expenses to provide for emergencies.

### WOMEN ON BOARDS OF MANAGEMENT OF HOSPITALS.

MISS LOUISA TWINING writes: As I have been considering and writing upon this subject for many years, I hope you will allow me to express an opinion upon it with regard to your article. I am unable to perceive the distinction drawn between boards of general hospitals and those for incurables, as the facts and reasons seem to me as strong in one class of institutions as the other. The fact remains the same that women are universally recognised as those who care chiefly for the sick, as well as being the acknowledged authorities on all domestic matters and duties; both of these claims exist in hospitals, yet women have no authority, and there is no one with power to represent them and the nurses on the official boards. I am not implying the slightest blame on the matrons, but I do say that there are cases when it is desirable that the women officers and patients should have the power of approaching and appealing to women in authority. The want of this privilege was stated and lamented by Mrs. Jameson in her admirable lectures on social questions 50 years ago, and she remarks on the absurdity of "lords and gentlemen" being the only persons with authority to visit and inspect the wards even of the hospitals for women and children! Surely there are at present sufficient educated women as inspectors and managers in numerous departments of work to render the remark as to "friction" and "amateurs" wholly irrelevant and unnecessary. Why are they more so than the non-medical men who at present chiefly form the board of management? At all events, we have facts and experience on our side in the results well-known in many country hospitals, which have long since adopted the plan and reforms universally with the most satisfactory results. I wish I could name all these, but the most recent instance is at Norwich, and I earnestly desire and hope to see, ere long, the same conviction prevail in London also.

### Appointments.

BASINGSTOKE UNION INFIRMARY.—Mrs. M. G. Charles has been appointed superintendent nurse. She was trained at Chorlton Union Hospital, Manchester, and has since been charge nurse at Leeds Union Infirmary, superintendent nurse at Warwick Union Infirmary, superintendent nurse at Eltham Union Infirmary, and head nurse at Battle Union Infirmary.

EASTVILLE WORKHOUSE, BRISTOL.—Miss Mary Ford has been appointed charge nurse. She was trained at Stapleton Infirmary, Bristol, where she has since been assistant nurse. She has also been charge nurse at Aston Infirmary and charge nurse at Christchurch Infirmary, Hampshire.

GREAT YARMOUTH INFIRMARY.—Miss Sarah Coggin Burd has been appointed charge nurse. She was trained at Romford Union Infirmary, where she has since been charge nurse.

HUDDERSFIELD SANATORIUM.—Miss Charlotte H. Richardson has been appointed charge nurse. She was trained at the Western Fever Hospital, London, and at the Royal Lancaster Infirmary, Lancashire. Since then she has done private nursing and been assistant nurse at the Borough Isolation Hospital, Leicester.

JAFFRAY HOSPITAL, ERDINGTON, BIRMINGHAM.—Miss Forence FitzHerbert has been appointed sister. She was trained at the Queen's Hospital, Birmingham, where she has since been sister-in-charge of the men's surgical ward, and casualty sister.

PRINCESS ALICE HOSPITAL, EASTBOURNE.—Miss Edith Peile has been appointed matron. She was trained at the Royal Surrey County Hospital, Guildford, and has since been charge nurse at the South-Western Fever Hospital, Stockwell, Queen's nurse at Aldershot and Dublin, and sister at the County Hospital, Guildford, and the Nursing Home, Blackwater Road, Eastbourne.

ROYAL HANTS COUNTY HOSPITAL, WINCHESTER.—Miss Carpenter-Turner has been appointed matron. She was trained at the Hospital for Sick Children, Great Ormond Street, London, and Leicester Infirmary, where she has since been assistant matron.

WALSALL WORKHOUSE INFIRMARY.—Miss Lucy Evans has been appointed charge nurse. She was trained at Aston Union Infirmary.

### Christmas Books.

The most striking feature of the books sent us for Christmas this year is the remarkable variety which they present. They are of all descriptions—suitable for little children, growing boys and girls, men and women; grave and gay; instructive and amusing; and they range in price from a penny to half a guinea.

T. C. and E. C. Jack.

Nothing more delightful in the way of fairy-stories has ever been produced than "In Fairyland," by Louey Chisholm (7s. 6d.). Several of the well-known favourites may be found among its pages, but attired in such fascinating new dresses that they have the charm of both old and new friends. The author of the stories affirms that the artist, Miss Catherine Cameron, "loved fairies, and often lived in fairyland." And there must be some truth in the affirmation, we fancy, for the pictures as well as the cover of white and gold, with its red-robed figure at the window, are simply beautiful. She will indeed be a lucky child who receives this book as a Christmas gift. Bunyan's "Pilgrim's Progress" (10s. 6d.) is a high-class edition, printed on thick paper with marginal references and headlines, handsomely illustrated with 30 pictures in colour by Mr. Byam Shaw. The artist has cleverly caught the spirit of the allegory.

Nelson and Sons.

This firm has even remembered the booklet to put in the children's stockings, and produced a nice little volume with six good coloured illustrations, and clear big letterpress, for the price of one penny. It is entitled "The Star in the East. Stories about Jesus." In "No End of Fun" (6d.), which is written in verse, there are pictures of children, on a blue, scarlet, or apricot ground, an original and attractive idea. "Robinson Crusoe" (1s.) has many brightly-coloured full-page illustrations, and a condensed story of the famous hero. "The Twins" (6s.) is certain to be one of the favourite books of the year. The verses are by John Shirley, and the illustrations in colour by John Hassall. Both are so apt, and the contrast between the exceeding goodness of Paul Montgomery Vincent Green, and the sad wickedness of Peter Augustus Marmaduke Green is so cleverly accentuated, that it is impossible to look over the pages without a hearty laugh. "The Children's Crusade" (3s. 6d.), by E. Everett Green, is a romantic story of the reign of King John, telling how the children of Christendom in 1212 sought to rescue the Holy Land from the Saracens, the stirring adventures of the hero among the Moors being most exciting. Mrs. Arthur in "Mother Maud" (3s. 6d.) tells a pleasant story of home and High School life suitable for girls between 12 and 16, and the account of how, in obedience to her parent's dying behest, Maud Charlton gives up the desire of her heart to "mother" the family is charmingly told. Equally welcome to damsels a little younger would be "The Girls of Cromer Hall" (2s.), by Raymond Jacberns. The rebelliousness of dear little Betty, the description of the school routine, its rules and regulations, are recounted in such an interesting way that, like Oliver Twist, the youthful readers would like to "ask for more." "Father M.P." (2s. 6d.), by Theodore Wilson Wilson, a book for boys and girls, deals with the doings of a large North-country family whose adventures include two accidents, a flood, and an exciting election, where, of course, Father is the successful candidate. The book, like most of those issued by these publishers, is well illustrated in colours. "The Water Finder" (1s.), by Julia Long, discloses the wonders of the divining rod and how the successful wielder of the wych-hazel is able to save a village from plague, and would be specially appreciated by thoughtful children. In "The Seymour Girls," by Mrs. Glasgow (9d.), the first attempts of three young girls at housekeeping during the absence from home of their parents is narrated in a bright and pleasant manner. Another capital girl's story is "The Little Heiress" (3s. 6d.), by Mrs. Bruce Clarke. May is the daughter of an American millionaire who comes to school in England, and becomes a general favourite. The story is naturally and freshly told. "Ringed by Fire" (5s.), is from the pen of E. Everett Green, whose books are always charming. This time she deals with the stirring events of the Franco-German war, introducing the siege and surrender of Metz. History and fiction are well combined.

Chatto and Windus.

The story of "Daventry's Daughter" (6s.), by Harold Bindgloss, will be appreciated by all who are fond of adventure. Told in a bright, attractive style, the scenes are chiefly laid in the Canaries and Morocco, and the narrator, a young merchant officer, is the hero of many strange experiences. His chief, Colonel Daventry, is the leader of the expedition which fails disastrously, but his daughter, the heroine, has a happy issue out of all her troubles. As "The Schemers" (6s.), is the first novel from the pen of Mr. Edward F. Harkins, he may be congratulated upon the humour and originality with which he describes certain phases of life in Boston. He has made a study of shop-girls in America, and has worked with skill a vein which authors on the other side of the Atlantic have almost let alone. "A Very Queer Business" (6s.), by William Westall, serves as the title of several characteristic stories by an author, who, during a long and busy life, attained a very fair measure of success. They vary in interest, but are sufficiently readable. The popularity of most of Sir Walter Besant's novels will long outlive the talented author, and though "The Alabaster Box" (3s. 6d.), is not one of his strongest productions, the new edition will find plenty of purchasers. A new edition of "The Mystery of Jamaica Terrace" (2s.), by Dick Donovan, will have numerous admirers among people who are fond of sensational and romantic detective stories.

Gay and Bird.

A new delightful book is "Rebecca" (6s.), by Kate Douglas Wiggin. It treats of New England life, and the heroine is a most quaint and winning child, whose original deeds and sayings are scattered throughout its pages. There is a touch of pathos underlying the bright humour of the volume. "The Affair at the Inn" (3s. 6d.) is the joint production of Kate Douglas Wiggin, Mary Findlater, Jane Findlater, and Allan McAulay, each of whom is responsible for the recounting of the incident from his or her own point of view. The book is "smart" from start to finish, with a laugh on every page.

Cassell and Co.

"Bo-Peep" (2s. 6d.) is certainly worthy of its second title "A Treasury for the Little Ones." The print is large and clear, the tales are thoroughly suitable, and there are numerous illustrations, coloured and otherwise. Most children will love "Cheepy the Chicken" (1s. 6d.). It is a delightful account of a small chicken who, owing to his discontent, is changed by the Queen—a white Pomeranian dog—into an elephant, a wallaby, and a rabbit successively, and so learns the lesson of making the best of things. "The Little Folks' Picture Album in Colours" (5s.), by S. H. Hamer, is full of good things to please the little ones, bright pictures, stories, and verse, and should be a popular present. Mr. Rider Haggard's "The Brethren" (6s.) has already passed through one edition. It deals in an exciting way with the contest fought out long ago between Cross and Crescent among the Syrian plains and deserts, introducing Saladin and his fierce Saracens, "The Old Man of the Mountain," Christian knights, and gentle ladies.

(*To be continued.*)

### Christmas Novelties.

BY OUR SHOPPING CORRESPONDENT.

### FOR CHRISTMAS TREES.

Fry's chocolates in an acceptable form for hanging upon Christmas trees, placing in Santa Claus' stocking, or in the lucky tub, are an acquisition when the difficulty of pleasing recipients of gifts of varying ages is before us. I notice some delightful little teapots and pretty little coloured straw baskets, full of the best chocolates, which can be secured for the expenditure of a few pence. Both have convenient attachments for hanging upon a Christmas-tree. For a larger expenditure Messrs. Fry have designed some very artistic boxes of bonbons, tied with pretty ribbands which cannot fail to please wherever they go.

The makers and venders of toys are learning that the children's ward is a factor to be reckoned with at Christmas. Consequently I have before me a special box of toys designed to find its way to the small patients spending a Christmas away from home. This box is a wonderful production for the money, for it contains ten attractive toys which the vendors, Messrs. Toler Brothers, of Savoy Corner, Thames Embankment, send to any one generously disposed towards small sick people, for the sum of one shilling. Toler's crackers and Christmas cards and ornaments for Christmas trees are also remarkably cheap; 100 pretty Christmas cards of really good quality are to be had also for the useful shilling.

### AT MESSRS. PENBERTHY'S.

I much admired a dainty blouse for evening or semi-evening wear, now being shown at Messrs. Penberthy's newly-extended premises at 388, 390, and 392 Oxford Street. It is in accordion-pleated mousseline de soie, trimmed with écru lace and herring-boned yoke, and is made in all colours as well as black and white. It is both pretty and tasteful, the accordion pleats giving it a very graceful appearance. The price is 29s. 6d., and this blouse, with many others for both morning and evening wear, may be seen in the beautifully-fitted ground-floor room, which is now devoted entirely to blouses and the various accessories—such as belts, collars, and ties—that are indispensable to our modern toilette. The firm is also showing very tasteful designs in hats, this department being presided over by a clever manageress from the Rue de la Paix; while another room is devoted exclusively to Colonial outfits, etc. Nurses and others wanting to choose useful presents for their friends, if unable to make a personal visit of inspection, should send for Messrs. Penberthy's price list of motoring and other gloves, hosiery, pocket-handkerchiefs, purses, etc., and as the postal despatch of this house is conducted with great promptitude, "shopping by post" can be performed with ease. Some time ago I drew attention to the unique Island Bouquet perfume of Mr. Edgar Dupuy of Guernsey. I am now glad to say that the difficulty of transport has been removed, for Messrs. Penberthy have now Island Bouquet on sale. It can be obtained nowhere else in London.

### CHRISTMAS GIFTS IN BOND STREET.

"What can I give?" is the question that will very soon be agitating our minds with pleasant anticipation of Christmas, and at Messrs. J. Harris and Sons, 25 Old Bond Street, the question may to a large extent be solved. The speciality of this firm is the beautiful flax material woven at their mills at Cockermouth, and embroidered in dainty threads. The latest production is a mixture of flax and silk, which combines the sheen of the one material with the durability of the other, and is of practically everlasting wear. I saw a dainty tablecloth of this texture in pale water-blue, on which oranges and orange-blossoms had been worked, with a particularly pleasing result. A very acceptable present for a nurse is a fitted writing-board. Pipe-racks, letter and post-card cases, motor and cycling books, hot water cosy-covers, medical receipt books, purses, bags, and a thousand and one dainty trifles, offer an endless variety of choice; nor must I forget to mention the pretty morning trays, made in the form of a violet-leaf, pansy, or lily of the valley, with doyley and china to match, which cannot fail to give pleasure to an invalid.

### CADBURY'S CHOCOLATES.

Messrs. Cadbury have, as usual, prepared their delicious chocolate and bon-bons in the most tempting fashion for Christmas givers. The most novel and tasteful covering perhaps, is a box closely imitating the popular old silver effect of what is called the new art. This is tied with pale blue riband, the bow of which secures a pretty little pair of bon-bon tongs. There are less ambitious cases of chocolate biscuits, milk chocolate, and all the specialities for which Cadbury is justly famed.

### THE BEST EAU DE COLOGNE.

Every year at this time I draw the attention of our readers to "4711" Eau de Cologne, and in doing so I reiterate the praises of this delightful perfume. I am not afraid that my opinion will be disputed. Every nurse who has invested in a bottle for herself or for a friend, will agree with me that its possession is always welcome, and its excellence beyond question. It can be obtained wherever perfumes are sold and at the depôt, 62 New Bond Street.

### WARM UNDERCLOTHING.

A useful gift can always count on a welcome at Christmas, as well as at any other season. I think that Dr. Lahmann's underclothing offers a great choice to intending givers. There is a little layette of a most attractive nature, including soft white garments of a nature to delight any mother's heart, whilst the sensible, well-fashioned clothing for men, opens a way out of the difficulty of finding something acceptable in this direction. A price list offers many suggestions, and this can be obtained from the Lahmann, Agency, 15 Fore Street, E.C.

### ROWNTREE'S CHRISTMAS NOVELTIES.

I have before me a charming box containing Rowntree's excellent Swiss milk chocolate. The box is artistically decorated with a design of blue gentians and green leaves, the two colours being carried out in the lettering, assisted by an outlining of gold. A mauve ribband enhances the original effect. Another novelty is a box of Marshmallow biscuits. These are delicions bonbons, shaped as small biscuits, and coated with chocolate.

### FOR THE TOILETTE.—ERASMIC SOAP.

Those who have used Erasmic soap will agree with me in thinking it one of the nicest soaps obtainable, and to those who have not yet made its acquaintance I can with confidence recommend it. The shilling boxes of three cakes make a useful and acceptable present. The soap is made at Warrington by Messrs. Crossfield, who are an English firm, doing much to promote the comfort of their employées.

### Notes and Queries.

**FOR REGULATIONS SEE PAGE 12.**

*Health Visitor.*

(72) (1) What course should one take to become a health visitor or inspector? (2) Could one be efficiently prepared by correspondence? (3) If not, are there any centres near Cheltenham? (4) Would the fact of one's being over 30 years of age be detrimental to a candidate seeking a post?—*C. S.*

(1) The nearest centre of instruction to Cheltenham is that of the Liverpool Ladies' Sanitary Association. The office and class rooms are at 8 Sandon Terrace, Upper Duke Street, Liverpool. The secretary will give you full particulars.

*Lady Doctor.*

(73) Can you kindly tell me where to write for information how to become a lady doctor?—*T.*

The Secretary, the London School of Medicine for Women, 8 Hunter Street, Brunswick Square, W.C., will give you full particulars.

*Hospital Training.*

(74) Will you kindly let me know whether a three years' training in a county general hospital containing 108 beds would be recognised as sufficient training to enable a nurse to enter the naval, military, or colonial nursing services?—*Dhurial.*

If the hospital in question is a recognised training school; but the number of beds is only one of several qualifications necessary. Write to the secretary of the service you wish to enter and inquire. The addresses and particulars of qualifications are all in "The Nursing Profession: How and Where to Train."

*Royal Infirmary, Edinburgh.*

(75) Will you kindly tell me if the professional status of the nurses trained at the Royal Infirmary, Edinburgh, is equal to that of the nurses trained at the leading London hospitals, or does the Royal Infirmary, Edinburgh, rank as a provincial hospital? And would a nurse with Edinburgh training be eligible for a post as sister in a London hospital?—*Nurse Mary.*

The Royal Infirmary, Edinburgh, is a recognised first-class training school, and a nurse in possession of its certificate is quite eligible for the post of sister in any London hospital.

*Registration.*

(76) Will you kindly let me know if a nurse, holding a certificate for three years' training at a county hospital containing 76 beds, will be able to register under the new code? If not, will it be a great disadvantage to her? Will it be troubling you too much to ask for the main points of this code also, and when you think it is liable to be brought into force?—*Helvetia.*

There is no code in force. Registration Bills have merely been under the consideration of a Parliamentary Committee.

*Open-air Sanatorium.*

(77) I should be glad to know of a sanatorium for consumption for a boy of 19 who has only just developed symptoms. The parents are quite poor, but his aunt (a nurse) could pay something.—*H. L. N.*

The Richmond House Sanatorium, Clare, Suffolk, receives a few men (early cases) 16s. a week. Or the Kelling Sanatorium, Holt, Norfolk, is for poor patients. But most of the large infirmaries now treat tuberculous patients on the open-air system. For list of these see "Burdett's Hospitals and Charities."

*Hospital Staff.*

(78) What would be a reasonable staff for 19 patients suffering from scarlet fever, of both sexes, from two and a-half years upwards, the patients being warded in two separate wards, and in the administration block? Present staff: one trained nurse, man as caretaker and messenger, and two inexperienced females to assist the nurse.—*Complications.*

A reasonable staff for 19 scarlet-fever patients of both sexes in two wards might consist of a matron or nurse-matron, or very responsible charge nurse acting as matron, two trained fever nurses, and two assistant nurses. Of the trained nurses, one should act as charge night nurse and one of the assistant nurses would also be needed for night duty. With regard to the services of the "two inexperienced females," we conclude that they are employed for menial work only, since no untrained woman should be responsible for any part of the nursing of so serious a disease as scarlet fever.

**Handbooks for Nurses.**

| | Post Free. |
|---|---|
| "The Nursing Profession: How and Where to Train" ... | 2s. 4d. |
| "The Nurses' Pronouncing Dictionary" ... ... ... | 2s. 0d. |
| "Nursing: its Theory and Practice" (Lewis) ... ... | 3s. 6d. |
| "Surgical Bandaging and Dressings" ... ... ... | 2s. 0d. |
| "A Complete Handbook of Midwifery" (Watson) ... | 6s. 4d. |

Of all booksellers or of The Scientific Press, Limited, 28 & 29 Southampton Street, Strand, London, W.C.

### For Reading to the Sick.

SPIRITUAL CHARACTER.

God's saints are shining lights: who stays
Here long, must passe
O'er dark hills, swift streames, and steep ways
As smooth as glasse:
But these all night
Like candles, shed
Theire beams, and light
Us into bed.

They are indeed our pillar-fires,
Seen as we go:
They are that Citie's shining spires
We travel to.
A sword-like gleame
Kept man for sin
First out; this beame
Will guide him in.

*H. Vaughan.*

We do not know the history of that character which is a birth of the Divine Spirit. That is the manifestation of which "thou canst not tell whence it cometh." It is indeed on account of this, and because its origin is lost in the mystery of God's spiritual creation, that the contemplation of it excites at once our awe and love. It is no earthly manufacture, and no copy or reflection of an outside pattern, but that it is an inspiration from the fountain-head of all life and goodness. And it is because we see this that we know it to be spiritual.

The character which has the unknown origin is itself a prophecy and presage of another world, because it seems made for it. Its source and its destination then are alike beyond our sight. We do not see that Great Spirit from which the sons of God derive their birth; we do not see that heavenly society of the spirits of just men made perfect toward which they are journeying. Whence they come and whither they go we see not; and that because they are born of the Spirit.—*J. B. Mozley.*

The power of mere activity is often overrated. It is not what the best men do, but what they are, that constitutes their truest benefaction to their fellow-men. The things that men do get their chief value, after all, from the way in which they are able to show the existence of character which can comfort and help mankind. Certainly, in our own little sphere, it is not the most active people to whom we owe the most. Among the people whom we know it is not necessarily those who are busiest, not those who, meteor-like, are ever on the rush after some visible change and work. It is the lives, like the stars, which simply pour down on us the calm light of their bright and faithful being, up to which we look and out of which we gather the deepest calm and courage. It seems to me that there is reassurance here for many of us who seem to have no chance for active usefulness. We can do nothing for our fellow-men. But still it is good to know that we can be something for them; to know (and this we may know surely) that no man or woman of the humblest sort can really be strong, gentle, pure, and good, without the world being better for it, without somebody being helped and comforted by the very existence of that goodness.—*Phillips Brooks.*

## Figures and Tables

**Figure f1:**
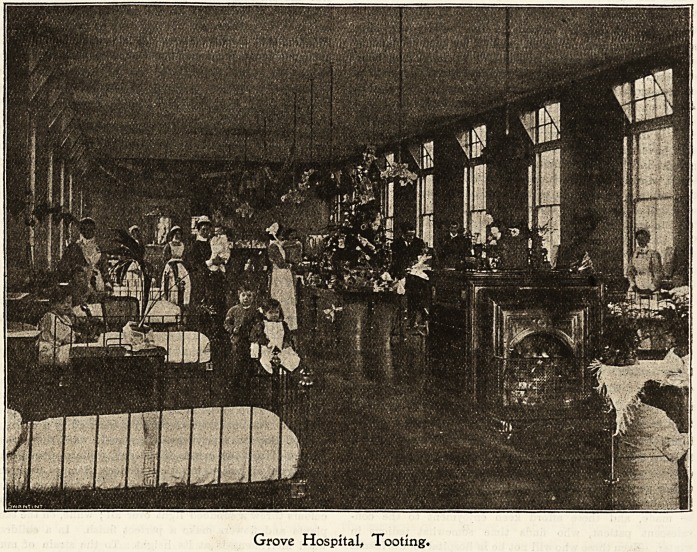


**Figure f2:**
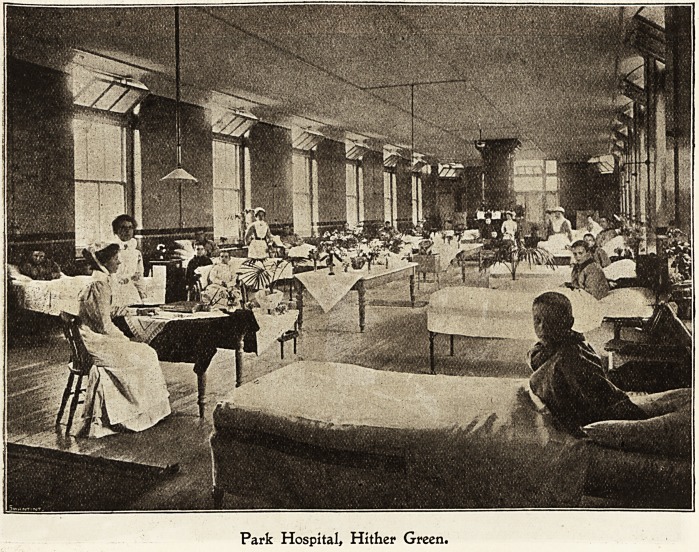


**Figure f3:**
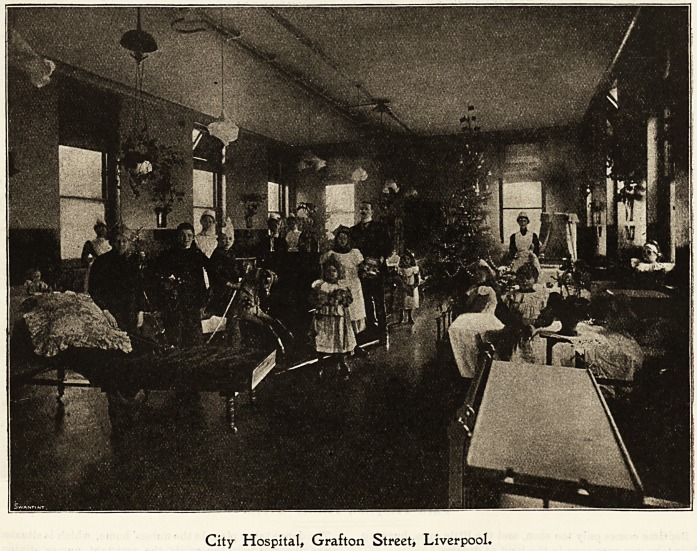


**Figure f4:**
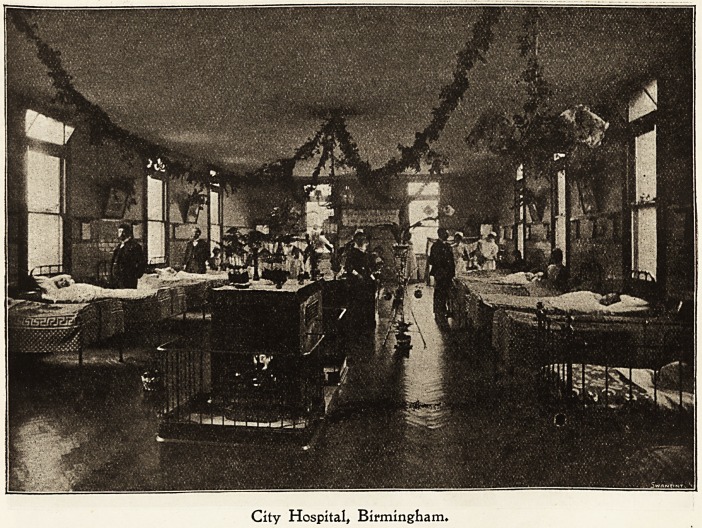


**Figure f5:**
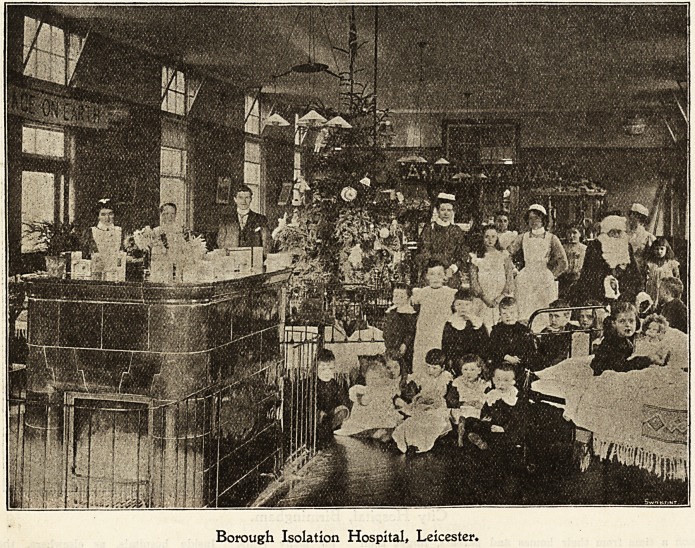


**Figure f6:**
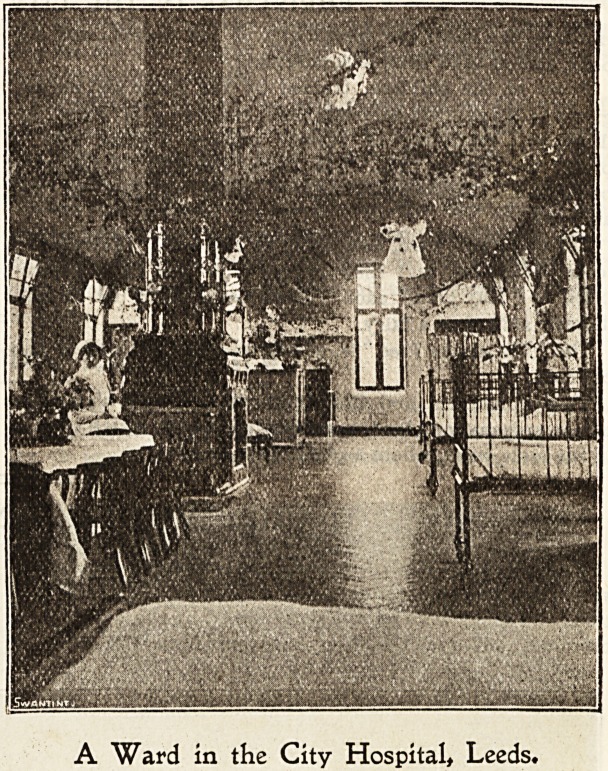


**Figure f7:**
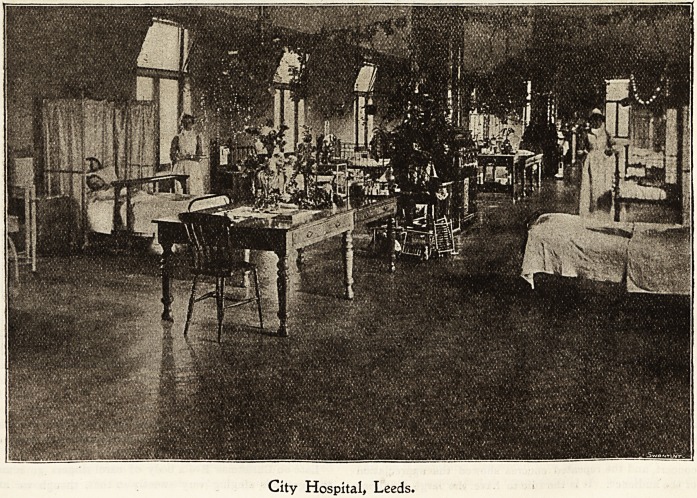


**Figure f8:**
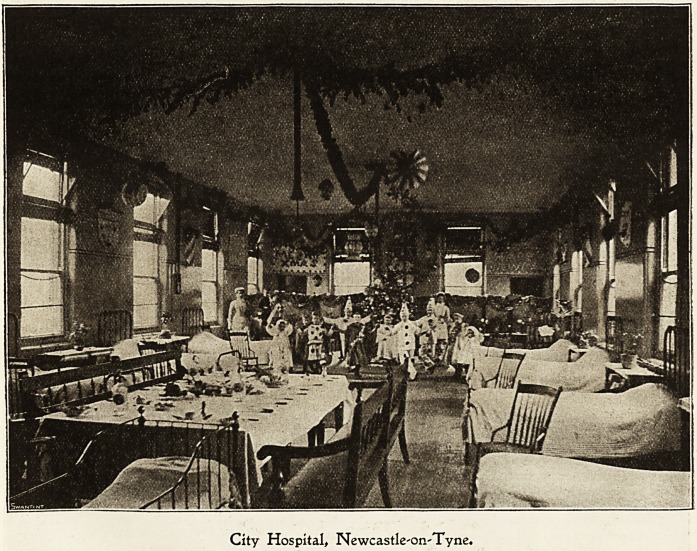


**Figure f9:**
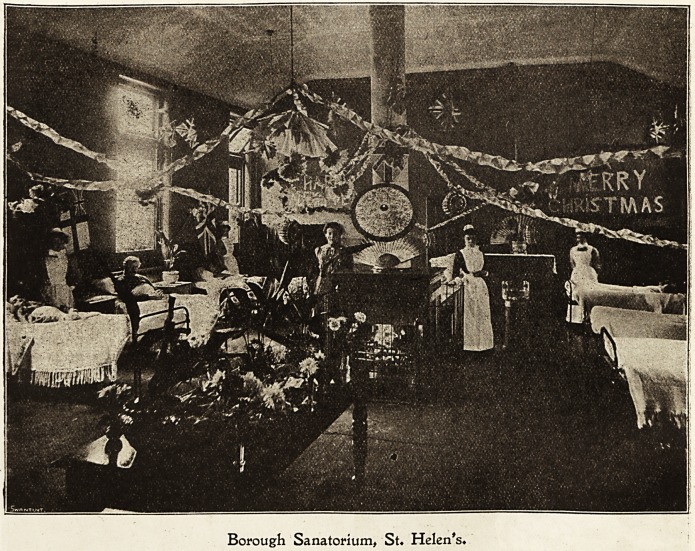


**Figure f10:**
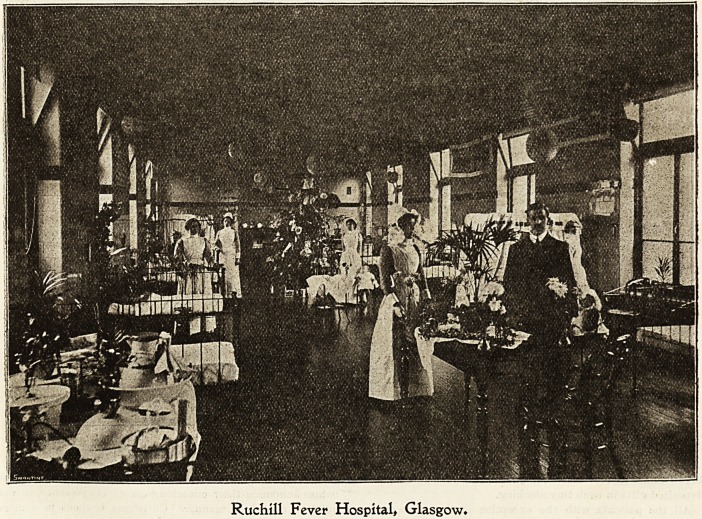


**Figure f11:**
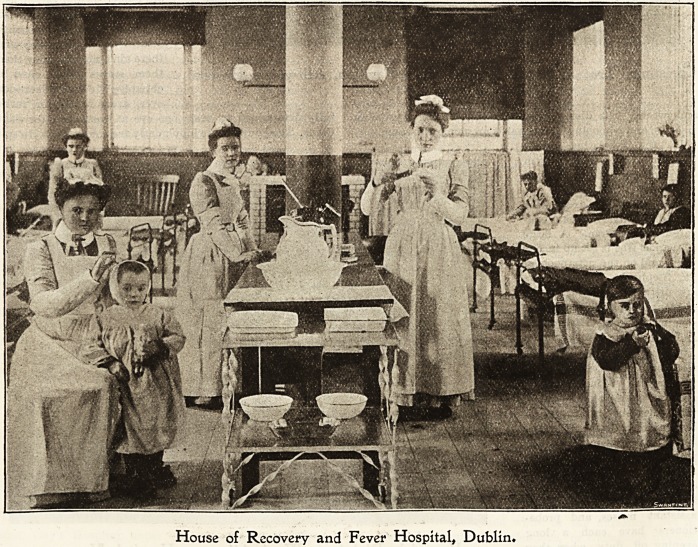


**Figure f12:**
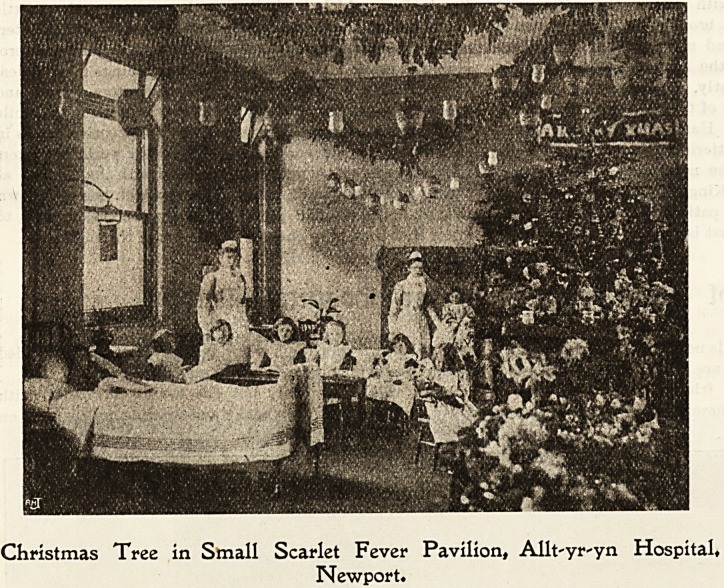


**Figure f13:**
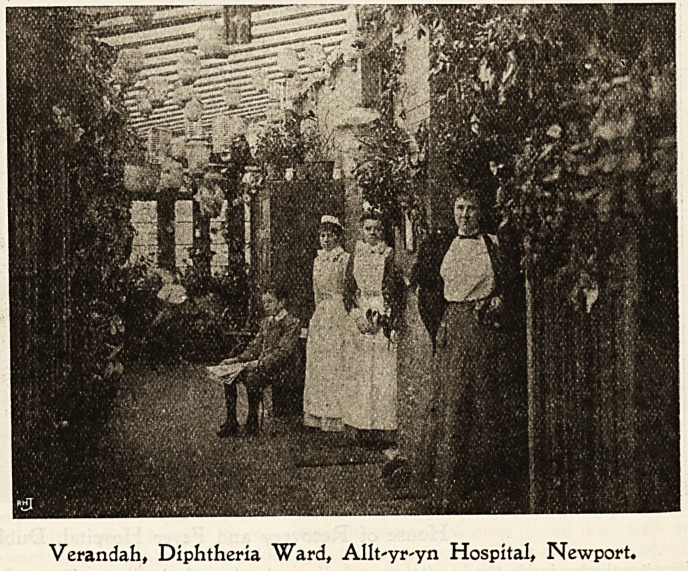


**Figure f14:**